# Drivers and epidemiological patterns of West Nile virus in Serbia

**DOI:** 10.3389/fpubh.2024.1429583

**Published:** 2024-07-17

**Authors:** Giovanni Marini, Mitra B. Drakulovic, Verica Jovanovic, Francesca Dagostin, Willy Wint, Valentina Tagliapietra, Milena Vasic, Annapaola Rizzoli

**Affiliations:** ^1^Research and Innovation Centre, Fondazione Edmund Mach, San Michele all’Adige, Italy; ^2^Department for Communicable Diseases Prevention and Control, National Public Health Institute “Dr Milan Jovanovic-Batut”, Belgrade, Serbia; ^3^Environmental Research Group Oxford Ltd., c/o Dept Biology, Oxford, United Kingdom

**Keywords:** mosquito, vector-borne, mathematical model, West Nile virus, *Culex*

## Abstract

**Background:**

West Nile virus (WNV) is an emerging mosquito-borne pathogen in Serbia, where it has been detected as a cause of infection in humans since 2012. We analyzed and modelled WNV transmission patterns in the country between 2012 and 2023.

**Methods:**

We applied a previously developed modelling approach to quantify epidemiological parameters of interest and to identify the most important environmental drivers of the force of infection (FOI) by means of statistical analysis in the human population in the country.

**Results:**

During the study period, 1,387 human cases were recorded, with substantial heterogeneity across years. We found that spring temperature is of paramount importance for WNV transmission, as FOI magnitude and peak timing are positively associated with it. Furthermore, FOI is also estimated to be greater in regions with a larger fraction of older adult people, who are at higher risk to develop severe infections.

**Conclusion:**

Our results highlight that temperature plays a key role in shaping WNV outbreak magnitude in Serbia, confirming the association between spring climatic conditions and WNV human transmission risk and thus pointing out the importance of this factor as a potential early warning predictor for timely application of preventive and control measures.

## 1 Introduction

West Nile virus (WNV) is a mosquito-borne virus, part of the genus Flavivirus which is rapidly becoming one of the most widespread emerging pathogens in Europe (1). It is maintained in an enzootic cycle between avian hosts and mosquito vectors, especially those belonging to the *Culex* genus (2). Mosquitoes acquire the infection after biting an infected bird and, after an incubation period, can then transmit the virus through subsequent blood meals. Mammals, including humans and equines, act as incidental dead end hosts in the natural transmission cycle, i.e., they cannot transmit the virus to mosquitoes (3). However, human-to-human transmission may occur through blood transfusions or organ transplantation (3). Although most of the human infections are asymptomatic, about 25% present symptoms such as fever and headache, and less than 1% develop severe neurological complications which can have a fatal outcome (3).

WNV is characterised by high genetic diversity. Phylogenetic analysis has identified at least eight evolutionary lineages of which WNV lineages 1 (WNV-1) and 2 (WNV-2) are the most widespread and pathogenic, causing continuous outbreaks in humans and animals around the world (4–7). More specifically, WNV-2 accounts for 82% of all WNV sequences detected in Europe so far, being found in 15 European countries (6). Between 2012 and 2023, about 6,700 human infections were recorded in the European Union, with large inter-annual differences (8).

Different environmental factors may influence WNV transmission (9). For instance, temperature affects mosquito biology: warmer conditions increase the developmental rate of immature stages but also decrease survival (10–12). Higher temperatures also decrease the incubation period of the virus in the vector population (13). Land use plays a key role as well at shaping not only mosquito dynamics but also composition of both vector and host populations (9). Additionally, counterfactual simulations suggest that the establishment of the current areas of WNV circulation in Europe can be largely attributed to climate change (14), thus highlighting the importance of climatic conditions for WNV circulation.

In Serbia, WNV infection in humans was confirmed for the first time in 2012 (15), and *Culex pipiens* mosquitoes are considered to be the major vector for WNV transmission in the country (16, 17).

In this study, we analyzed and modelled WNV transmission patterns in Serbia between 2012 and 2023. We applied a previously developed modelling approach (18) aiming to quantify epidemiological parameters of interest and to identify the most important environmental drivers of the force of infection (FOI) in the human population in the country.

## 2 Methods

Serbia is a country 88,499 km^2^ wide located in Central Europe with about 6.6 million inhabitants. WNV human case-based data were provided by the Serbian National Public Health Institute and include date of disease onset, importation status (i.e., whether the infection was acquired in Serbia or abroad), age group, gender and the probable place of infection at the NUTS (Nomenclature of territorial units for statistics) 3 level (19). We restricted our analysis to probable and confirmed autochthonous human cases with known place of infection.

As in Marini et al. (18), we denote by *h_y,i_*(*w*) the number of recorded WNV human cases with region *i* as place of infection with symptoms onset occurred during week *w* of year *y* (*w*∈{1, …, 52}, *y*∈{2012, …, 2023}), by *H_y,i_* the whole time series, i.e., $H_{y,i}=\overset{52}{\underset{w=1}{\cup }}h_{y,i}\left(w\right)$ , and by Σ_y,i_ the total number of cases with place of infection, identified as *i*, recorded during year *y*, i.e., $\Sigma_{y,i}=\overset{52}{\underset{w=1}{\sum }}h_{y,i}\left(w\right)$ .

We modelled observed epidemiological curves using the FOI-model proposed in Marini et al. (18). We assumed *h_y,i_*(*w*) coming from a Poisson distribution with average $\underset{t\in T_{w}}{\sum }N_{i}\cdot \lambda_{y,i}\left(t\right)$ , where *λ_y,i_*(*t*) denotes the WNV FOI (i.e., the rate at which susceptible humans acquire the infection) in region *i* and year *y* at day *t*, *T_W_* represents the set of days in week *w* and *N_i_* is the number of inhabitants of the region.

The number of inhabitants, also stratified by age group, for each considered NUTS3 region was retrieved from the Eurostat database (20).

As in Marini et al. (18), we assumed that the FOI for region *i* and year *y* could be modelled through the density function of a normal distribution, i.e.

$$\lambda_{y,i}\left(t\right)=c_{y,i}\cdot \frac{1}{\sigma_{y,i}\sqrt{2\pi }}e^{-\frac{1}{2}{\left(\frac{t-\mu_{y,i}}{\sigma_{y,i}}\right)}^{2}}$$

Where *μ_y,i_* and *σ_y,i_* represent, respectively, the average and standard deviation of the distribution and *c_y,i_* is a magnitude rescaling factor.

Hence, *μ_y,i_* indicates the Julian day of year *y* for which *λ* reaches its maximum in region *i*, *σ_y,i_* provides an estimate for the length (in days) of the epidemiological season and finally *c_y,i_* is a measure of the FOI magnitude in that year and geographical area. These three parameters were estimated by matching the generated epidemiological curve to the observed data through a maximum likelihood approach (considering only series with $\Sigma_{y,i}\geq 5$ , i.e., NUTS3 regions and years with at least 5 cases). We denote with M, S and C the estimated distributions of *μ_y,i_*, *σ_y,i_* and *c_y,i, respectively._* Additional modelling details can be found in Marini et al. (18).

We then quantified through linear models (see below) the relationships between the response variables S, M and C with a set of 6 covariates of potential interest defined as in Marini et al. (18):

1) η*(i)*: the total percentage of Corine Land Cover (CLC) labelled as urban or agricultural area. This measure can be interpreted as a proxy for the anthropogenic impact on the region *i*. Proportions of land cover classes for each spatial unit were derived from the 2018 CLC data inventory (21).
2–3) T_spring(y,i)_ and T_summer(y,i)_: respectively the average spring (April–May) and summer (June–July) Land Surface Temperature (LST) recorded in region *i* during year *y*. Monthly 5 km resolution Land Surface Temperature (LST) was derived from the MODIS (Moderate Resolution Imaging Spectroradiometer) MOD11c3 and VNP21A1D datasets (22, 23).
4–5) P_spring(y,i)_ and P_summer(y,i)_: respectively the cumulative spring (April–May) and summer (June–July) precipitation occurred in region i during year y. Monthly 5 km resolution cumulative precipitation data were derived from downscaled daily ECMWF (European Centre for Medium-Range Weather Forecasts) ERA5-Land datasets and downloaded from the Climate Data Store (24).
6) *ε(i)*: the fraction of people older than 65 years living in region *i* (20).

We first computed a full Linear Model which can be represented by the following equation:

$$Y\sim \eta +T_{\mathrm{spring}}+T_{\mathrm{summer}}+P_{\mathrm{spring}}+P_{\mathrm{summer}}+\varepsilon$$

Where *Y* can either be S, M or log(C) (we normalised the C distribution by log-transforming it).

We checked for potential collinearity among explanatory variables by computing Variance Inflation Factors (VIFs) (25). We then computed all possible submodels and selected as best the model with the lowest Akaike Information Criterion (AIC) score and whose coefficients were all statistically significant. Model assumptions were verified by checking residuals distributions and by plotting residuals versus fitted values and versus each covariate in the model (25).

All analysis was carried out in R v4.3.1 (26) using libraries “tidyverse” (27) and “MuMIn” (28). The R functions used to perform the model fit can be found at https://github.com/giomarini/epiCurve-repository.

## 3 Results

**Figure 1:**
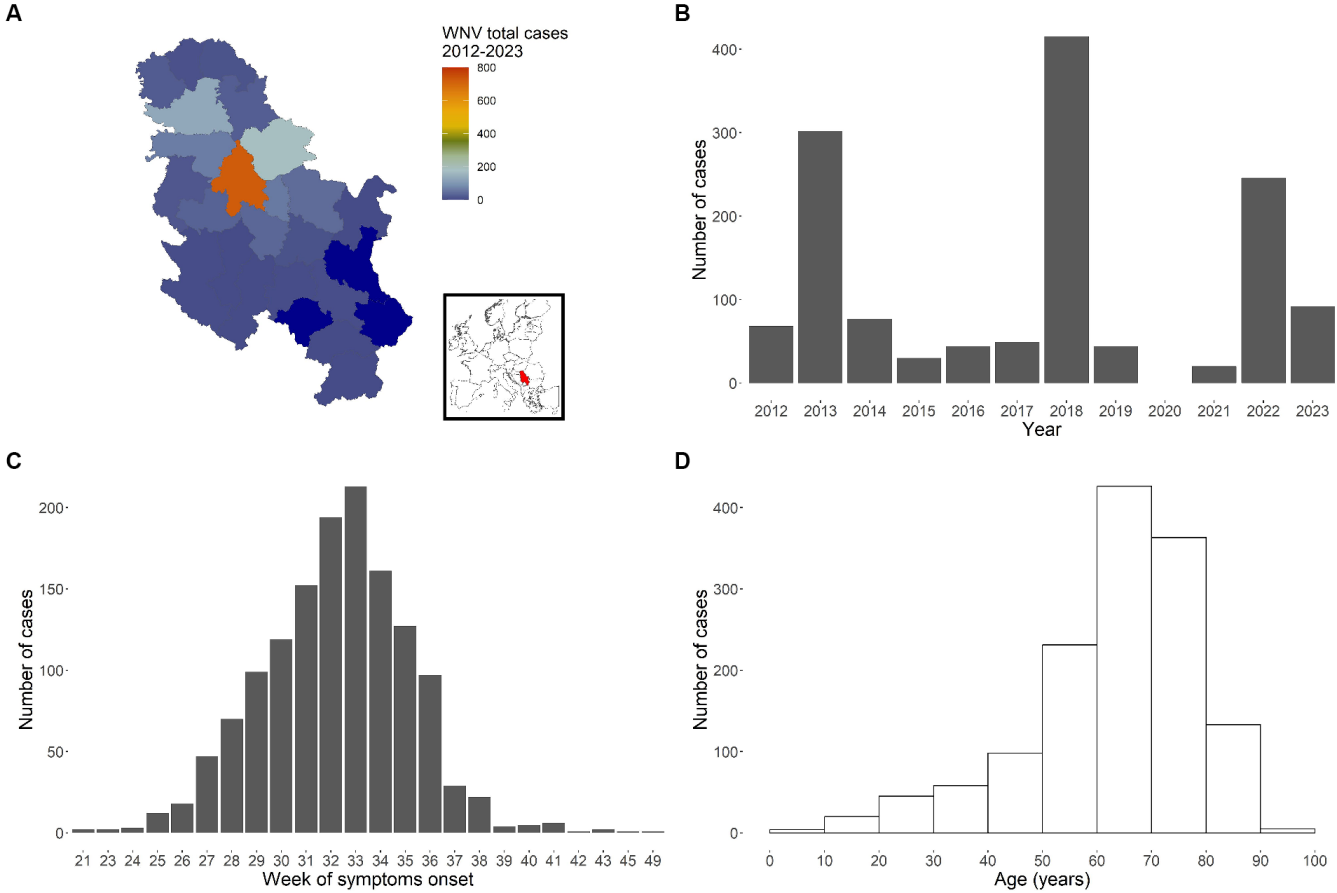
WNV cases recorded in Serbia between 2012 and 2023. Total number of cases by administrative area (NUTS3 level) (**A**, with inset map highlighting Serbia), by year **(B)**, by week of symptoms onset **(C)** and by age group **(D)**.

Between 2012 and 2023, a total of 1,387 autochthonous human cases were reported from 23 different NUTS3 regions (Figure 1A), all belonging to WNV-2 (29). Cases were mostly males (852, 61.4%). The lowest number of infections was observed in 2021 (20 cases), and the highest (415 cases) in 2018. For the year 2020 data collection was limited due the Covid-19 pandemic: 23 suspected WNV infection cases were notified, but they were not confirmed by laboratory analysis and they were not included in the analyses.

The cumulative epidemiological curve (total number of cases per week of symptoms onset across all years and regions, Figure 1C) clearly shows a peak around the 33rd week of the year (first half of August). Finally, Figure 1D reports recorded cases by age, thus highlighting the observed higher likelihood for older people to develop symptoms and thus being notified to the surveillance system.

We applied our FOI modelling approach to 51 epidemiological curves *H_y,i_* with on average 25.1 total cases (min = 5, max = 213, sd = 39.2). The majority of the considered epidemiological curves belonged to 2018 (10 curves), 2022 (9 curves) and 2013 (8 curves), when the three largest outbreaks occurred (Figure 1B).

**Figure 2:**
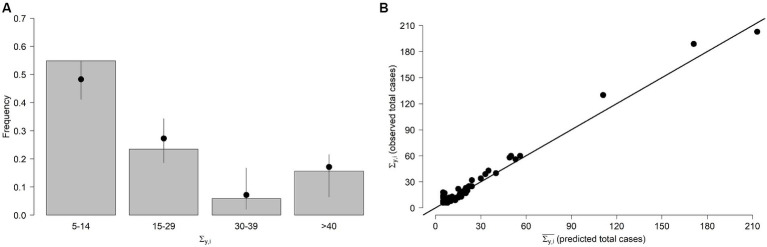
FOI model. **(A)** Frequencies of the stochastically predicted and observed Σ*_y,i_* (total number of WNV human cases recorded during year *y* in region *i* ). Bars: observed Σ*_y,i_*. Points and lines represent average and 95% quantiles of the frequencies of the stochastically predicted values, respectively. Values are shown aggregated by group. **(B)** Predicted $\left(\bar{\Sigma_{y,i}}\right)$ and observed $\left(\Sigma_{y,i}\right)$ total number of WNV human cases for each region and year.

We generated 100 stochastic realisations for each *H_y,i_* prediction and compared the frequencies of the observed and predicted values. More specifically, the number of human cases with day of symptoms onset *t* expected for region *i* and year *y* were drawn from a Poisson distribution Pois(*λ_y,i_(t)*). We found our model fits well observed cases as 97.4% of the simulated total number of weekly cases lie within the 95% Confidence Interval (CI) of model predictions. From Figure 2A, which shows observed and predicted frequencies for the total yearly number of WNV cases Σ*_y,i_*, we can also note a very good agreement between the two quantities. Finally, there was a very good correlation (Pearson correlation coefficient = 0.94) between the predicted ( $\bar{\Sigma_{y,i}}$ ) and observed ( $\Sigma_{y,i}$ ) values (Figure 2B), with an average squared error $E{\left(\Sigma_{y,i}-\bar{\Sigma_{y,i}}\right)}^{2}=35.7$ .

**Figure 3:**
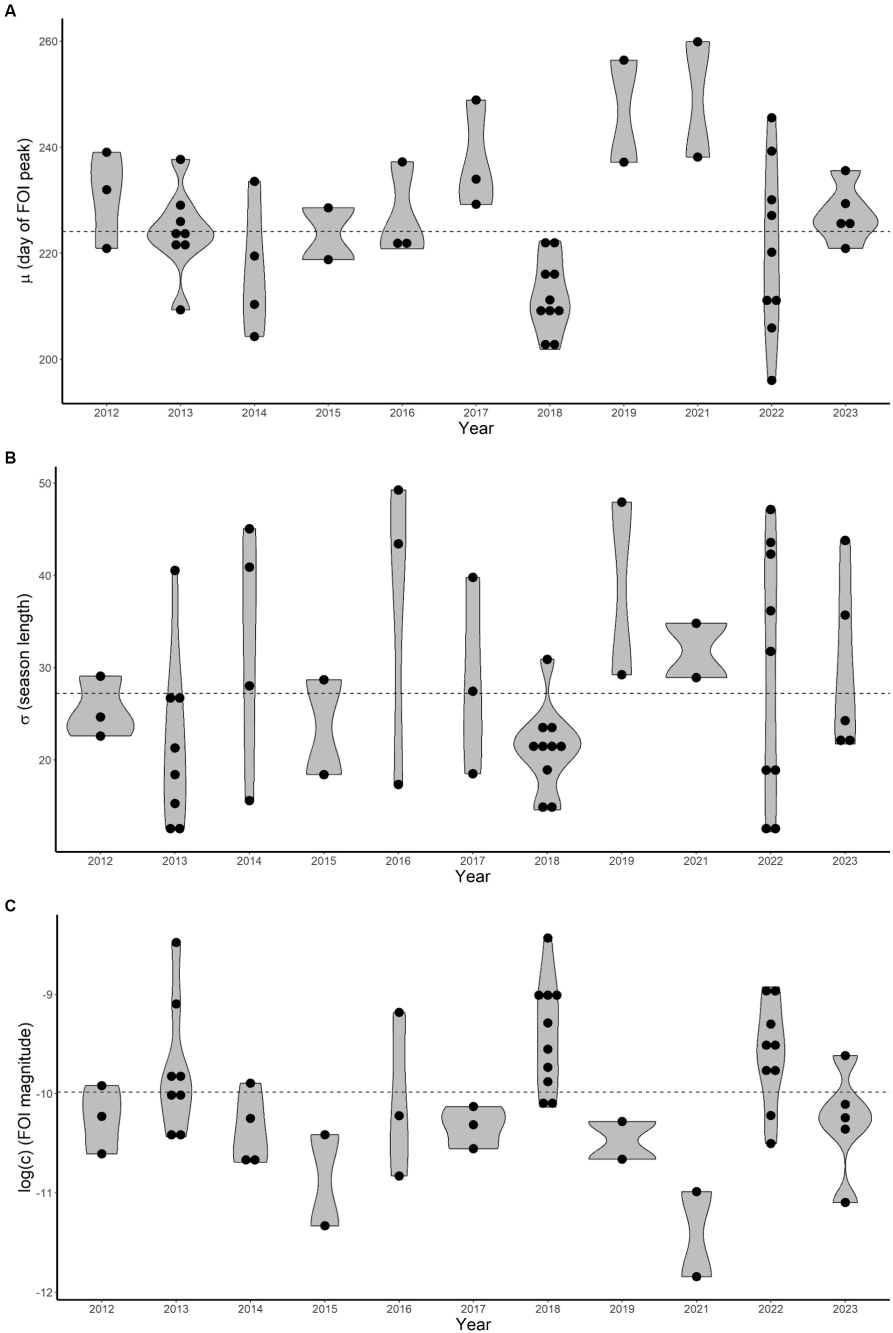
Estimated average (*μ*), standard deviation (*σ*) and magnitude (*c*, log-transformed) distributions (violin plots, **A**–**C** respectively) for each year. Dots represent estimated values for each epidemiological curve. Dashed horizontal lines are average values computed overall years.

The estimated distributions of the three free FOI-model parameters (*c*, μ, σ) are characterised by a substantial temporal heterogeneity, as shown in Figure 3. The WNV FOI peaked later in 2019 and 2021 and was higher in 2018 and 2022.

The average parameter μ (indicating when the FOI reaches its maximum) ranged between 196 and 260 (July 15–September 17) with a mean value of 224 (August 12) and a 95% Confidence Interval (CI) lying within 202–254 (July 21–September 11). The average for σ (length of the epidemiological season) is about 27 days (95%CI 13–48). Finally, the rescaling parameter *c* (FOI magnitude) is on average 5.89∙10^−5^ (95%CI 1.28∙10^−5^-1.89∙10^−4^).

It is interesting to note that a shorter epidemiological season does not straightforwardly imply fewer human infections as σ values are mostly estimated to be below average for 2013 and 2018, when the FOI magnitude was greater.

**Figure 4:**
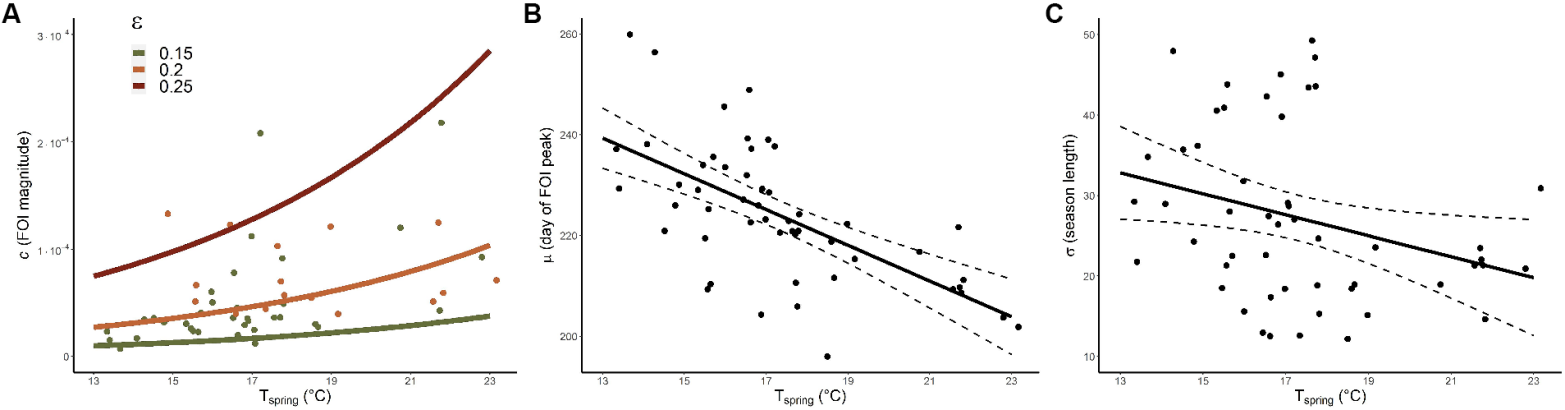
Model predictions. **(A)** Model predictions (lines) for *c* (FOI magnitude) conditional to *T_spring_* (average spring temperature) and ε (fraction of inhabitants older than 65 years). Green (orange) dots represent FOI-model values with associated ε belonging to the 0.15–0.2 (0.2–0.25) interval. **(B,C)** Model predictions for μ (day of the year when the FOI reaches its maximum) and σ (length of the epidemiological season) respectively; continuous and dashed lines provide average and CIs respectively, dots represent FOI-model values.

As VIFs were all below 3 we did not discard any explanatory variable from the full statistical models (25). The best model for *C*, whose coefficients are reported in Table 1, included two covariates (*R*^2^ = 0.4). As depicted in Figure 4A, we found that FOI magnitude is positively associated with spring temperature (*T_spring_*) and is estimated to be greater in areas with higher proportions of older adult people (ε). The best model for *M* (*R*^2^ = 0.4) included only the average spring temperature (see Table 1 and Figure 4B) indicating that warmer springs correspond to an earlier timing of the incidence peak. Finally, *T_spring_* was found to be the only significant negative predictor also for *S*, meaning that epidemiological seasons are shorter with warmer springs (*R*^2^ = 0.09), see Figure 4C.

**Table 1:** Estimates, standard errors, *t* values and *p*-values of the parameters of the best models for C, M and S.

*Y*	Parameter	Coefficient estimate	Standard error	*t* value	*p*-value
*C*	Intercept	−16.295	1.268	−12.855	<0.001
	ε	20.228	6.042	3.348	0.002
	*T_spring_*	0.134	0.033	4.109	<0.001
*M*	Intercept	285.433	10.783	26.471	<0.001
	*T_spring_*	−3.545	0.617	−5.746	<0.001
*S*	Intercept	49.740	10.404	4.781	<0.001
	*T_spring_*	−1.302	0.595	−2.186	0.034

## 4 Discussion

In this study we analysed and modelled WNV human transmission patterns in Serbia between 2012 and 2023. We applied a previously developed modelling framework (18) to investigate observed transmission patterns and identify the main environmental drivers of the FOI (the rate at which susceptible individuals acquire the infection) in the country. The deployed modelling framework, which aims at quantifying the WNV FOI using a limited number of parameters, was selected because it requires only data on recorded human infections. If more detailed data, such as entomological collections providing both mosquito abundance and WNV prevalence, are available, then other types of models might be developed to explicitly consider mosquito population dynamics and WNV transmission between vector and host populations (30, 31).

We found that spring temperatures are crucial at shaping WNV epidemiology, confirming previous findings demonstrating the importance of spring conditions in Europe for enhancing WNV circulation (18, 31–34). Warmer conditions are associated with an earlier peak of the FOI and a shorter epidemiological season but also with a larger FOI magnitude. Interestingly, estimated model coefficients for *T_spring_* for the FOI magnitude (*c*, 0.134) are consistent with previous estimates at continental level [0.142, see (18)], suggesting spring temperature exerts a comparable effect at both spatial scales.

At European scale we found that infection peak tends to be earlier when summer temperature is higher (18), whereas for Serbia we found a similar association but with spring conditions. Indeed, summer temperatures did not seem to significantly affect WNV transmission in the country, probably because most of the amplification phase had occurred previously. As warmer conditions might amplify virus transmission (i) by increasing mosquitoes’ biting rate (11) and (ii) the host-to-vector transmission probability (35, 36) and (iii) by shortening the mosquito viral incubation period (13), it is likely that favourable conditions during spring have a cascading effect later in the year, increasing human transmission risk. Consistently with our previous findings at European level (18), the FOI is estimated to be greater in areas with a higher number of older adult (age > 65 years) people. This association is unsurprising since age is one of the main risk factors for developing severe symptoms upon infection (37).

We found that model parameters are not significantly associated with precipitation-related variables, similarly to findings of our previous modelling efforts at continental level (18). Even though precipitation could indirectly influence the transmission dynamics of WNV by affecting mosquito breeding habitats and mosquito abundance, the direct effect of precipitation on WNV transmission may vary depending on local ecological and environmental conditions (9, 38).

Interestingly, land cover did not seem to significantly affect epidemiological parameters, whilst previously we found a negative association between η (total combined percentage of CLC labelled as urban or agricultural area) and the FOI magnitude *c* at European level (18). This result could depend on the narrower area under study and the lower variability of η across Serbia, with values ranging between 51 and 93% whilst at continental level we found it to vary between 22 and 97% (18). Our findings are consistent with recent phylodynamic models suggesting that WNV-2 is attracted to areas characterised by high crop and vegetation density, livestock cultivation, and urbanisation (6).

It is important to note that other factors could play an important role in shaping WNV circulation as well. For instance, a high avian immunity at the beginning of the epidemiological season, due to the previous year WNV circulation, might prevent pathogen transmission (39). Other climatic variables not explicitly considered in our model, such as drought or winter temperature might influence mosquito population dynamics and consequently WNV transmission as well (33, 40, 41).

We remark that the considered time series was incomplete as data collection in 2020 was limited because of the Covid-19 pandemic. This might also explain the low number of recorded cases in 2021, which however was consistent with other European countries (8).

As our modelling approach requires only human data, which are usually routinely collected by European health authorities, it might be easily applied to investigate the transmission of WNV or other vector-borne pathogens in other areas of interest. In fact, our proposed modelling framework is not specifically designed for WNV only, but it could be applied to any other vector-borne disease for which the FOI has a seasonal pattern and does not depend on the number of infectious humans such as tick-borne encephalitis or Usutu (18).

Our results point out the importance of weather anomalies at the beginning of the mosquito breeding season, which might amplify WNV circulation with a cascading effect later in the season. As previously highlighted in Farooq et al. (32), spring climate parameters should be given priority when developing climate-related WNV early warning systems.

## Data availability statement

The data analyzed in this study is subject to the following licences/restrictions: the data that support the findings of this study are available from the Serbian National Public Health Institute but restrictions apply to the availability of these data, which were used under licence for the current study, and so are not publicly available. Requests to access these datasets should be directed to mitra_drakulovic@batut.org.rs.

## Ethics statement

Ethical review and approval was not required for the study on human participants in accordance with the local legislation and institutional requirements. Written informed consent from the patients/participants or patients/participants legal guardian/next of kin was not required to participate in this study in accordance with the national legislation and the institutional requirements.

## Author contributions

GM: Conceptualization, Data curation, Formal analysis, Methodology, Writing – original draft. MD: Data curation, Resources, Writing – review & editing. VJ: Data curation, Resources, Writing – review & editing. FD: Data curation, Methodology, Writing – review & editing. WW: Data curation, Resources, Writing – review & editing. VT: Writing – review & editing. MV: Data curation, Resources, Writing – review & editing. AR: Conceptualization, Writing – review & editing.
